# Implementation of a nurse-led overdose prevention site in a hospital setting: lessons learned from St. Paul’s Hospital, Vancouver, Canada

**DOI:** 10.1186/s12954-022-00596-7

**Published:** 2022-02-05

**Authors:** Elizabeth Dogherty, Carlin Patterson, Marilou Gagnon, Scott Harrison, Jocelyn Chase, Jill Boerstler, Jennifer Gibson, Sam Gill, Seonaid Nolan, Andy Ryan

**Affiliations:** 1 St. Paul’s Hospital, Providence Health Care, 1081 Burrard Street, Vancouver, BC V6Z 1Y6 Canada; 2 Canadian Institute for Substance Use Research, University of Victoria, 2300 McKenzie Avenue, Victoria, BC V8N 5M8 Canada; 3 Providence Health Care Ethics Services, 1081 Burrard Street, Vancouver, BC V6Z 1Y6 Canada; 4 Department of Medicine, University of British Columbia, 2775 Laurel Street, Vancouver, BC V5Z 1M9 Canada; 5 BC Centre on Substance Use, 400-1045 Howe Street, Vancouver, BC V6Z 2A9 Canada

**Keywords:** Addiction, Injection drug use, Harm reduction, Hospital, Overdose, Overdose prevention, Substance use

## Abstract

**Objectives:**

In May 2018, St. Paul’s Hospital (SPH) in Vancouver (Canada) opened an outdoor peer-led overdose prevention site (OPS) operated in partnership with Vancouver Coastal Health and RainCity Housing. At the end of 2020, the partnered OPS moved to a new location, which created a gap in service for SPH inpatients and outpatients. To address this gap, which was magnified by the COVID-19 pandemic, SPH opened a nurse-led OPS in February 2021. This paper describes the steps leading to the implementation of the nurse-led OPS, its impact, and lessons learned.

**Methods:**

Four steps paved the way for the opening of the OPS: (1) identifying the problem, (2) seeking ethics guidance, (3) adapting policies and practices, and (4) supporting and training staff.

**Results:**

The OPS is open between 10:00 and 20:00 and staffed by two nurses per shift. It is accessible to all patients including inpatients, patients in the Emergency Department, and patients attending outpatient services. Between February 1, 2021 and October 23, 2021, the OPS recorded 1612 visits for the purpose of injection, for an average weekly visit number of 42. A total of 46 overdoses were recorded in that 9-month period. Thirty-seven (80%) required administration of naloxone and 12 (26%) required a code blue response.

**Conclusions:**

Due to the unique nature of our OPS, we learned many important lessons in the process leading to the opening of the site and the months that followed. We conclude the paper with lessons learned grouped into six main categories, namely engagement, communication, access, staff education and support, data collection, and safety.

## Introduction

Canada is in the midst of an ongoing overdose crisis. Between January 2016 and March 2021, more than 22,000 people died from an overdose [1]. The provinces of British Columbia, Ontario, and Alberta account for 90% of those deaths [1]. Overdose deaths are now responsible for the plateauing of the national life expectancy [2]. In British Columbia, where it is estimated that close to 6 people die every day from an overdose [3], life expectancy is now decreasing [4]. The toxicity of the drug supply combined with prohibition and criminalization has been and remains an important driver of this crisis. Between 2018 and 2021, 87% of the postmortem toxicology reports conducted by the British Columbia Coroners Service [3] detected fentanyl. This percentage remained stable in 2021, but fentanyl prevalence has risen in the illicit drug supply [3] and opioid overdoses involving benzodiazepines and sedatives such as xylazine have increased—resulting in more complex overdoses [5].

In April 2016, British Columbia declared a public health emergency in response to an increase in overdoses and overdose deaths. Since then, the number of supervised consumption services in British Columbia has gone from two to approximately thirty. This rapid scale-up was made possible by grassroots efforts which led to the opening of “pop-up” overdose prevention sites (OPS) and  compelled the Minister of Health to issue a ministerial order allowing overdose prevention sites to be opened (and integrated into existing services) without completing the traditional application process with Health Canada to be formally recognized supervised consumption sites. In British Columbia, OPS are typically staffed by peers with lived and living experience of substance use who provide first-aid interventions to prevent and respond to overdoses. They also act as a point of contact to connect clients with services and care.

In May 2018, St. Paul’s Hospital (SPH) in Vancouver became the first hospital in Canada to open an OPS, the Thomus Donaghy OPS, an outdoor peer-led supervised consumption service operated in partnership with Vancouver Coastal Health and RainCity Housing. At the end of 2020, the OPS moved to a new location (a 7 min walk from the hospital), which created a gap in service for both hospital inpatients and outpatients. To address this gap, which was magnified by the COVID-19 pandemic, SPH opened a nurse-led OPS in February 2021 and became the second hospital in Canada to offer supervised consumption services in an acute care setting [6, 7]. This paper describes the steps leading to the implementation of the nurse-led OPS, its impact, and lessons learned.

## Methods: process leading to the opening of the OPS

Four steps paved the way for the opening of the OPS: (1) identifying the problem, (2) seeking ethics guidance, (3) adapting policies and practices, and (4) supporting and training staff.

### Step 1: identifying the problem

SPH serves the downtown eastside and downtown core of Vancouver, with a focus on historically marginalized populations. One in six patients admitted to SPH’s 450 beds has an active substance use disorder and referrals to SPH’s Addiction Medicine Consult Team have increased by 228% over the past 5 years. Guided by a comprehensive policy framework outlining the philosophy for care for patients who use substances, SPH has implemented a wide range of innovative and progressive harm reduction programs and services over the years. When the Thomus Donaghy OPS opened in 2018, the objective was to prevent overdoses and overdose deaths within and around the hospital. Inpatients were using the OPS, but overdoses within the hospital continued to occur. Between January and December 2020, 21 code blues were called for suspected overdoses in the hospital. These code blues resulted in increased pressure on acute care teams and moral distress for staff who recognized the presence of an overdose risk but could not direct patients to a safer consumption space within the hospital. This problem coupled with the peer-led OPS having been relocated and the need to limit movement of patients during the COVID-19 pandemic led to the opening of the nurse-led OPS on February 1, 2021.

### Step 2: seeking ethics guidance

Providence Health Care’s (PHC) Ethics Services team was consulted to review whether it was ethically justifiable for SPH to open and operate an OPS. Organizational ethics is the discipline concerned with the principles and standards by which an organization operates. It concerns finding the “right” way to respond to complex challenges and opportunities. PHC leadership requested formal ethical analysis and recommendations regarding the opening of an OPS, as it was recognized that doing so could significantly impact the lives of patients and affect PHC’s reputation as an organization.

Following a detailed ethical analysis, the Ethics Services team determined that opening and operating an OPS is ethically permissible. In light of the risks of harm—that is, the degree of severity and certainty of harms related to overdose and overdose deaths—the ethics team identified proportionate rationale and clear ethical justification to move forward with an OPS. With an overarching goal to save lives and engage patients in care, harm reduction strategies (including an OPS) can form a comprehensive program of services for people who use substances. Findings from the ethical analysis also emphasized that an OPS should not be viewed as promotion of an individual’s substance use disorder, but rather an extension of compassionate care in alignment with the founding mission of SPH. Consult recommendations were endorsed and adopted by PHC Senior Leadership.

### Step 3: adapting policies and practices

Because of its long history of providing care to people who use substances, SPH has an extensive set of policies and guiding documents related to substance use (see Table 1). To implement the nurse-led OPS, some operational changes were made to policies and procedures such as removing bans on the possession of some substances and providing options for patients to store their substances safely (e.g., bedside safes). Additionally, a document outlining the standard operating procedures and duties of OPS staff was created. The existing decision support tool (DST) for nursing management of suspected overdose was adapted for the OPS setting because nurses would be witnessing patients injecting as opposed to finding them already overdosing in another location (e.g., bathroom, hospital room). For example, we adapted the DST to allow nurses to administer additional doses of naloxone and wait for the naloxone to have an effect prior to escalating care by initiating a “code blue” (emergency response).

**Table 1:** St. Paul’s policies and guiding documents by area of focus

Philosophy of care for patients and residents who use substances		
Inpatient (acute) care	Overdose	Opioid-related treatment
Managing substance use and harm reduction	Overdose management	Methadone
Alcohol withdrawal	Dispensing naloxone kits	Injectable opioid agonist treatment
Managed alcohol program	Code blue	Buprenorphine/naloxone
Cannabis withdrawal	Oxygen therapy	Buprenorphine/naloxone induction kits
Non-medical cannabis		IV fentanyl for withdrawal management
Tobacco management		
Unsafe sharps plan		
Bedside safes		
Search: room/belongings		

### Step 4: supporting and training staff

Positions to staff the OPS were advertised via both internal and external processes. Applications were invited from qualified licensed practical nurse (LPN) candidates with a minimum of 1-year experience working with people who use substances. Those hired attended mandatory training covering topics such as substance use disorders, trauma-informed practice, violence prevention, overdose response, drug testing, and harm reduction practices and interventions. They were also required to complete a shadow shift in a community OPS. Peers were involved in the training and provided staff with advice on supporting patients and best practices when working in an OPS. When the OPS opened, an announcement was sent out to the entire organization and nursing leadership was asked to disseminate the information to their clinical teams. Information on the OPS was also added to the standard nursing orientation to ensure that new employees know about the service to provide information to patients.

## Results

### Description of the site

**Fig. 1:**
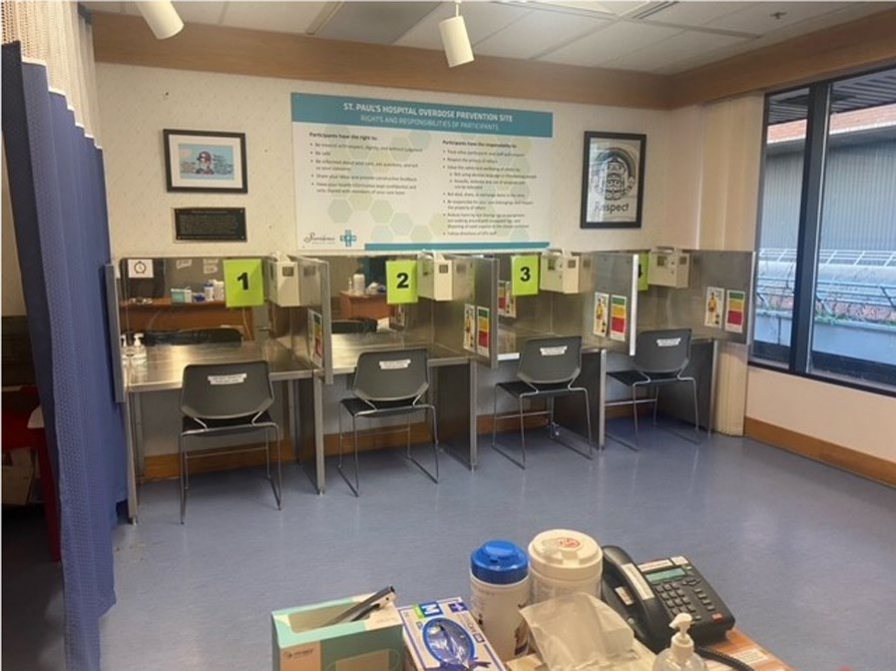
Picture of the OPS

The OPS opened in February 2021 (see Fig. 1). The site is staffed by two LPNs per shift who have access to a Registered Nurse for clinical support if needed. The site is open to all patients including inpatients, patients in the Emergency Department, and patients attending outpatient services. Visitors are permitted if they are supporting a patient but otherwise are directed to a community OPS. The site is open between 10:00–20:00 and is limited to injection use. Patients bring their pre-obtained (mostly non-prescribed) substances to inject in 1 of 4 consumption booths. After use, patients are encouraged to stay for a 10–15 min monitoring period to ensure no adverse reactions occur. In the event of a severe/complex overdose (i.e., not reversed by oxygen and naloxone), a “code blue” is initiated and Intensive Care Unit (ICU) staff respond. In order to maintain anonymity, staff from the inpatient units are only notified of patients’ OPS visit if an overdose occurs at the site. OPS staff are able to provide safer use education, drug testing and sterile harm reduction supplies. They also offer harm reduction education and supplies for patients who plan on using their medically necessary intravenous lines to inject.

### Recorded visits, overdoses, and code blues

**Fig. 2:**
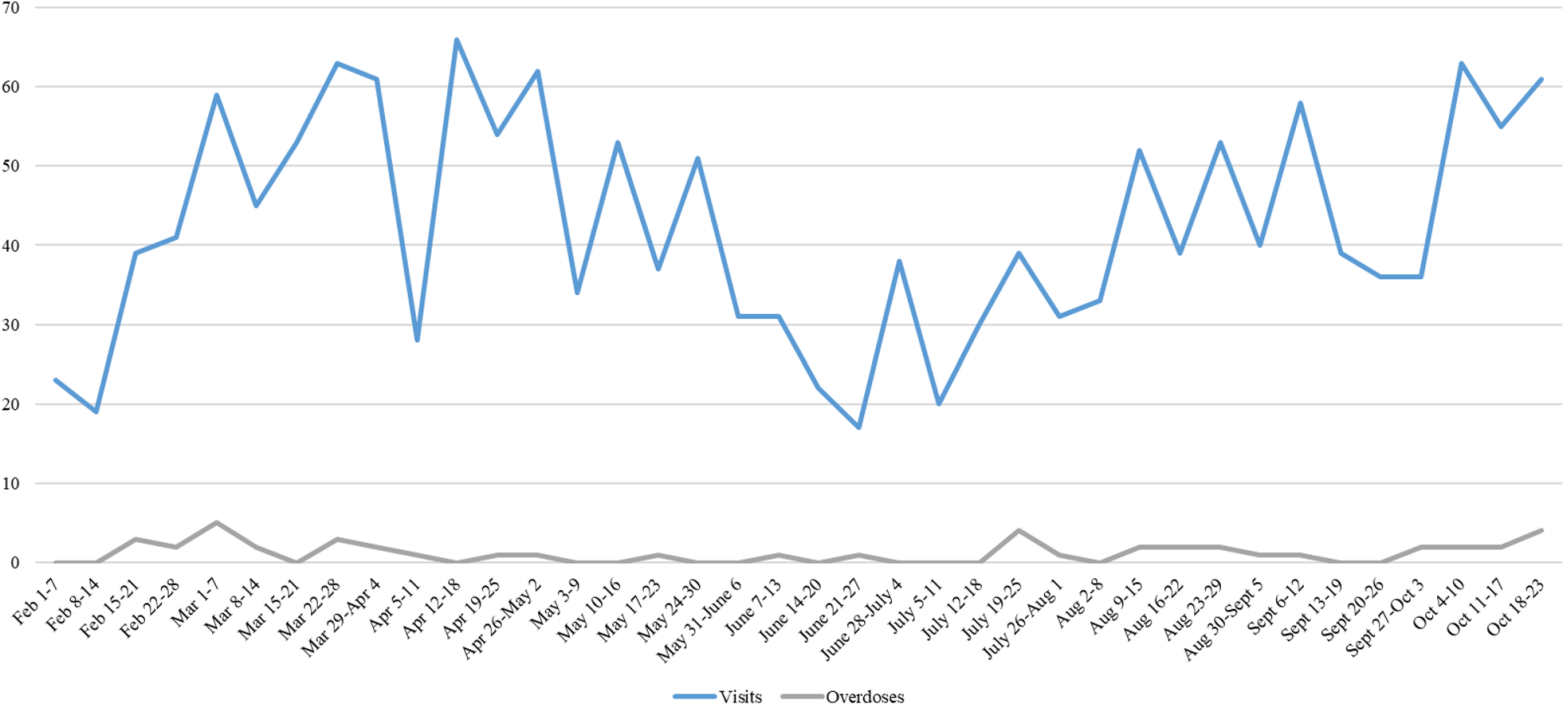
Weekly visits and overdoses

**Fig. 3:**
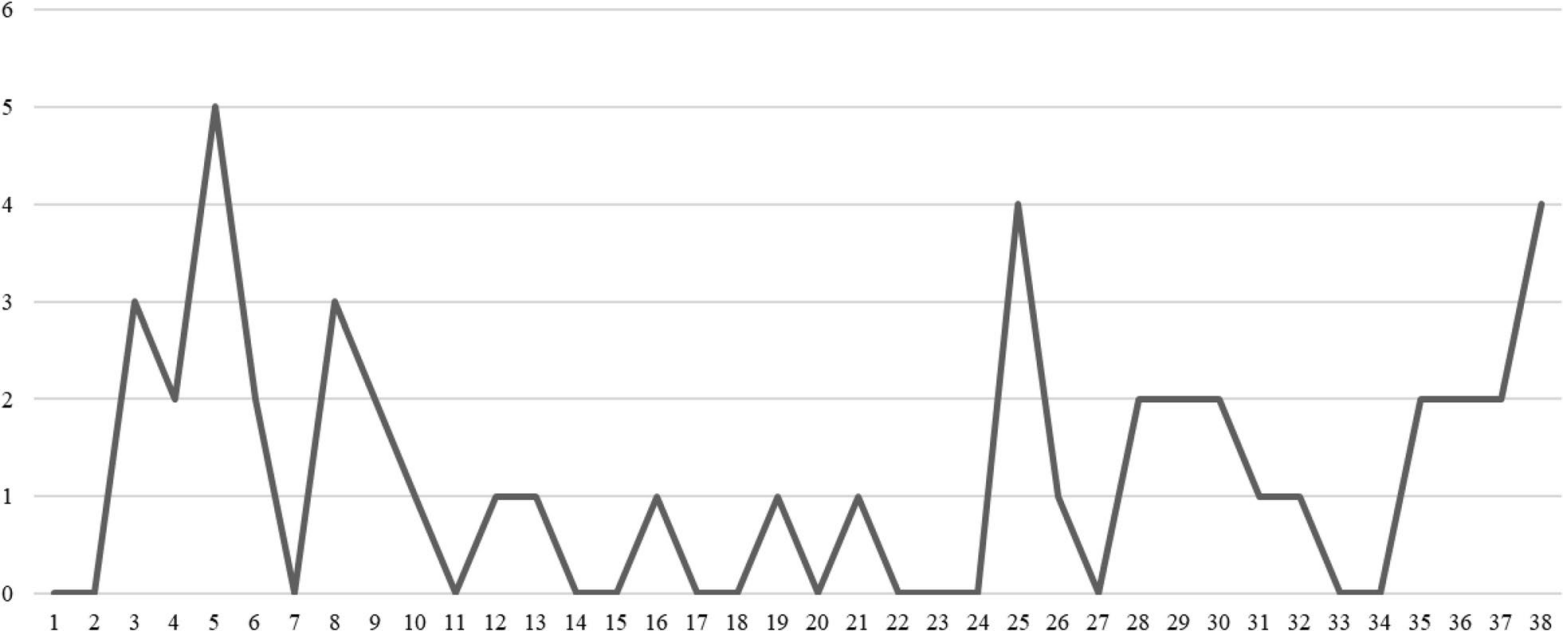
Number of overdoses per week (total of 38 weeks)

Between February 1, 2021 and October 23, 2021, the OPS recorded 1612 visits for the purpose of injection, for an average weekly visit number of 42. An overview of the visits and overdoses per 7-day period is shown in Fig. 2. A total of 46 overdoses were recorded in that 9-month period. A breakdown of overdoses per week is shown in Fig. 3. Here, it is important to note that overdoses are recorded when clients require interventions such as oxygen and naloxone administration. Depending on clinical presentation, some patients who require stimulation and monitoring of oxygen saturation may also be recorded as overdoses and some may not. Of the recorded 46 overdoses, 37 (80%) required naloxone administration and 12 (26%) required a code blue response. Based on the current data, it is challenging to determine if the OPS had an impact on the number of code blues called for overdoses in the hospital. The main reason being that the year before and after opening the nurse-led OPS are not comparable. For example, when the Thomus Donaghy OPS was opened, staff called 911 instead of code blues, which means data is lacking pre-2021. Furthermore, the increased toxicity of the drug supply during COVID-19 has resulted in a rise of overdoses and overdoses deaths in British Columbia. This could explain the documented rise in overdose-related code blues at SPH in 2021 (*n* = 27). We know that nearly 45% of those code blues happened in the OPS, which speaks to a positive impact, however as many code blues occurred when the OPS was closed. Potential reasons for the remaining code blues include lack of awareness that an OPS was available onsite, inability to leave unit to come to the OPS, and inhalation (not injection) as the primary method of consumption.

## Discussion

Due to the unique nature of our OPS, we learned many important lessons in the process leading to the opening of the site and the months that followed. We grouped the lessons learned into six main categories, namely engagement, communication, access, staff education and support, data collection, and safety.

### Engagement

Engaging all stakeholders at every step of the process is particularly important. Based on our experience, this includes the nursing staff and leadership directly involved with the OPS, members of the SPH emergency response team who might be called to the OPS, the ICU multidisciplinary team, nursing staff and leadership on the inpatient units, and site security. For example, after the first few emergency responses at the OPS, we realized that more collaboration was needed with the ICU team. To address this, we met with ICU leadership and gathered useful feedback on our operating procedures and overdose response protocol.

### Communication

To ensure that all SPH staff know about the OPS, multiple rounds of communication are needed. After our initial round of communication when the site opened, we distributed postcards with a site map and information about the OPS to SPH staff. Our goal was to provide staff with a simple document they could give to patients who may benefit from the OPS (e.g., patients who inject) and encourage them to proactively inform patients to avoid the risk of unwitnessed overdoses in the hospital. We also included this information in staff orientations. Nonetheless, overdoses continued to occur during OPS opening hours which suggests a need for more communication and ongoing reminders.

### Access

Our goal was to facilitate access to the OPS by using a number of strategies, including providing anonymity, protecting confidentiality, creating a welcoming and judgment-free environment, and adopting a harm reduction approach that also extends to patients who inject using their intravenous line. We recognized the importance of having a robust plan in place in the event that OPS staff needed to communicate with unit staff following an overdose—and to communicate this plan to patients using the OPS. This was particularly important because some patients were wary about their unit staff becoming aware that they were using the OPS. Finally, opening hours remain an access issue. In the absence of 24/7 access, overdoses have continued to occur onsite, primarily once the OPS is closed for the day.

### Staff education and support

Education and support were required for staff at the OPS *and* on the units. We realized early on that, besides knowing about the OPS, unit nurses required education and support regarding follow-up care for patients brought back to the unit after an overdose. We provided in-services and a one-page written resource (a practice pointer) outlining post-overdose best practices. We also engaged with unit-level nursing leadership, including charge nurses and educators who could provide shoulder-to-shoulder guidance for nursing staff as they provide follow-up care. Education and support for OPS staff were quite different in part because education was provided upon hiring. OPS staff needed support because they worked in isolation compared to regular units, did not have management onsite, relied on outside staff for help in case of emergencies, and often found themselves in the middle of patients and unit staff. As such, we saw a clear need for additional resources to support the OPS staff, such as designated staff who could assist with transporting patients to and from the OPS. The OPS staff also benefited from debriefing following overdoses and code blues. We used debriefing sessions to adapt our operating procedures and overdose response protocol.

### Data collection

Our data collection system tracked the number of visits, overdoses, frequency of naloxone administration, number of code blues, and number of patients who used their intravenous line. While keeping access to the site as low-barrier as possible, gathering more information related to the patients visiting the OPS would be helpful. Additionally, we see the need for dedicated time, resources, and funding to explore the patient and staff perspectives, track outcomes more closely, and document the impact of the OPS, such a possible reduction in patient initiated discharges. As such, we would recommend developing an evaluation plan along with establishing the OPS.

### Safety

We came across four main safety-related issues, namely location, flow, protocol, and staffing model. The location of our OPS is ideal from an access standpoint because it is situated near the cafeteria and outdoor garden patio, but it can complicate the work of staff because no acute care unit is located on that floor. It is also situated in a busy area with only one available emergency door. Initially, the lack of space at the OPS to relax after injecting resulted in patients staying at the site for hours. To ensure flow at the site, we had to implement a limit of 30–45 min but it remains challenging to enforce. We grappled with questions related to staff safety, including when to call security, what system to use, and so forth. We opted to use a button that activates a code white, security checks, and provide two types of phones that staff can use. We are also actively working to increase peer involvement at the site.

In conclusion, our experience of opening a nurse-led OPS in acute care offers an important example of hospital-based harm reduction and valuable lessons that can inform the scaling up of this intervention in other acute care settings. While more research and data are needed to assess the clinical impact of the OPS, we already know anecdotally that it is making a meaningful difference for patients and staff. Our OPS model presents some limitations, such as operating hours and injection-only services, but it an excellent starting point to ensure that patients who use substances are provided with a safer space when they receive hospital-based care.

## Data Availability

Not applicable.

## Abbreviations

OPS: Overdose prevention site
ICU: Intensive care unit
SPH: St Paul’s Hospital
